# Emergence of hierarchical organization in memory for random material

**DOI:** 10.1038/s41598-019-46908-z

**Published:** 2019-07-18

**Authors:** Michelangelo Naim, Mikhail Katkov, Stefano Recanatesi, Misha Tsodyks

**Affiliations:** 1 Department of Neurobiology, Weizmann Institute of Science, Rehovot, 76000 Israel; 2 Department of Physiology and Biophysics, University of Washington, Seattle, WA 98195 USA

**Keywords:** Neural encoding, Long-term memory

## Abstract

Structured information is easier to remember and recall than random one. In real life, information exhibits multi-level hierarchical organization, such as clauses, sentences, episodes and narratives in language. Here we show that multi-level grouping emerges even when participants perform memory recall experiments with random sets of words. To quantitatively probe brain mechanisms involved in memory structuring, we consider an experimental protocol where participants perform ‘final free recall’ (FFR) of several random lists of words each of which was first presented and recalled individually. We observe a hierarchy of grouping organizations of FFR, most notably many participants sequentially recalled relatively long chunks of words from each list before recalling words from another list. Moreover, participants who exhibited strongest organization during FFR achieved highest levels of performance. Based on these results, we develop a hierarchical model of memory recall that is broadly compatible with our findings. Our study shows how highly controlled memory experiments with random and meaningless material, when combined with simple models, can be used to quantitatively probe the way meaningful information can efficiently be organized and processed in the brain.

## Introduction

Free recall of randomly assembled lists of words is a long-standing paradigm for studying human memory that produced a great amount of experimental observations^1–9^ and theoretical models^10–15^. One of the critical observations made over the decades of research concerns the issue of performance, i.e. the number of words that people can recall from lists of various lengths. It was observed that recall performance grows sublinearly with the list length, which means that even for lists of moderate lengths, people cannot recall most of the words presented to them^1–9^. These observations stand in a stark contrast to much better recall of meaningful texts, such as stories or poems. One plausible explanation for this difference could be in the fact that meaningful texts exhibit various degrees of organization that makes them easier to remember and recall. For example, a story may contain distinct episodes that relate to each other in multiple logical chains that give rise to its ‘meaning’ and makes it more memorable^16–18^. Often speakers introduce organization to the material to be communicated in order to improve its retention; one of the most prominent organizational strategies is grouping (the parceling of information into smaller parts). Pausing at appropriate places while speaking, allows listeners to divide the speech into meaningful parts^19^. Several studies of chunking during free recall were pursued. In some of them, chunking was imposed by the presentation protocol, such as e.g. increasing the time interval between the chunks^20–23^, while in others spontaneous chunking was observed^21,24–27^. The typical size of a chunk was related to the working memory capacity (see e.g.^28^), which was also found to be positively correlated to free recall performance^29^.

Several studies reported that repeated recall of random lists resulted in increased chunking^16,30,31^. In our recent analysis of the data of M. Kahana where each list was presented once, a small fraction of participants developed strong chunking, accompanied by a substantial improvement of performance, up to perfect recall of full lists of 16 words^32^. However, the overall extent of chunking is very moderate which makes the analysis of its effects on performance quite difficult, in particular a vast majority of participants did not exhibit chunking even after extensive practice.

A highly elaborate model of temporal clustering in both serial and free recall was developed in^24^, based on earlier models^12,20,33,34^. The main idea of this model is a hierarchical representation of temporal context that incorporates episodic clustering into distinct groups of individual items and also serial position of items within each group. The recall of each item is preceded by the retrieval of a group context, which in turn is triggered by group-specific cues and control elements. The main focus of the model is induced or spontaneous chunking in single list recall, characterized by the appearance of short clusters of 3–4 subsequently presented words. Another influential model of clustering, called Context Maintenance and Retrieval (CTM) is proposed in^14^. This model generalizes the earlier Temporal Context Model^12^ to include the possibility that different memory items are grouped into distinct sources (e.g. words presented aurally vs visually). The model accounts for experimentally observed interplay between two different types of clustering: a temporal one based on presentation position of different items, and source clustering.

In this contribution, we chose to focus our attention on the paradigm of final free recall (FFR), which could be considered as a strong version of induced chunking. In this paradigm, participants recall several lists in a single daily session, in the end of which they are asked (with no prior warning) to recall the words of all the lists in an arbitrary order. FFR was studied in several previous publications (see e.g.^35–39^). Most of these studies concerned the differences between temporal organization of recall within lists as assessed by a classical serial position curve^2^, for both single list recall and FFR. In some studies, similar organization was reported for both cases, characterized by primacy and recency effects^35^, while other studies, involving bigger number of longer lists, reported within-list ‘anti-recency’ in FFR^38,39^. It was also reported that words from the lists presented towards the end of the session were more likely to be recalled (list recency) and between-the-list transitions tend to be between lists that were presented one after another (list contiguity)^36,37^.

We conjectured that FFR should exhibit strong grouping over the lists, because each list was presented and recalled individually in the same session. We also wanted to elucidate the possible effects of grouping on the FFR performance. Indeed over-the-list grouping was observed in^36^, but only the average length of within-list clusters was reported (2.6 words our of 10 words in each list) and no analysis of its effect on FFR performance was presented. Our analysis not only uncovered a highly significant overall degree of grouping in FFR, but also demonstrated the great diversity of it across participants. Moreover, we found a very strong positive correlation between grouping and FFR performance, thus reinforcing the crucial role of temporal organization of information in episodic memory, in a precisely quantifiable way.

To elucidate the possible mechanisms of grouping and its effect on FFR performance, we developed a highly reduced version of hierarchical recall models of^14,24^. It generalizes our previous model that successfully accounted for power-law scaling in free recall of single lists^32,40,41^ and includes some of the features that are similar to^42^. To reduce the complexity of the model, we did not include any mechanisms for generating within-list temporal organization which resulted in significantly fewer free parameters than in the previous theoretical studies. We found that the model accounts well for both overall degree of grouping and its diversity, as well as the correlation between grouping and FFR performance in terms of the number of words recalled.

## Results

### Grouping over lists, induced by presentation protocol

**Figure 1:**
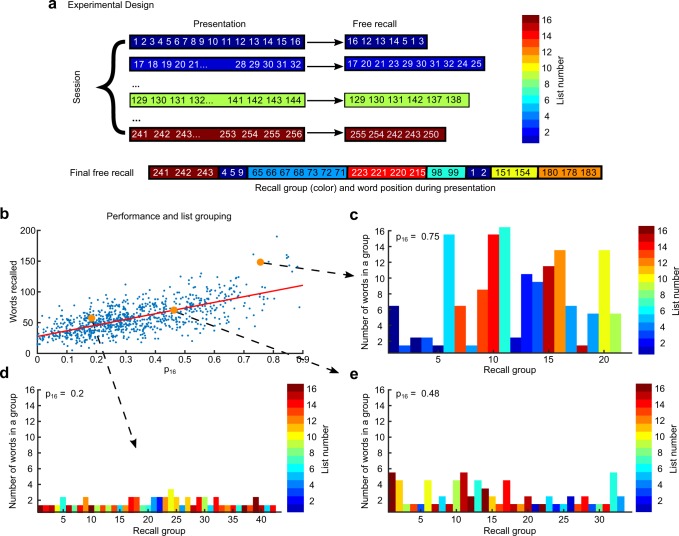
Experimental design and list-grouping results. **(a)** Experimental design - each day 16 lists were presented to human participants (colored lines on the left, with numbers representing serial presented position of the word during the day, and color representing the list number from blue to brown with color code presented in the right), after presentation of a list participants recalled as many words as they could (colored lines in the middle with a serial position of recalled words); at the end of some days participants performed final free recall (FFR), where they recalled as many words presented during the day as they could (bottom line with each recalled word colored according to the presentation list). **(b)** Number of recalled words during FFR vs. grouping measure *p*_16_ (see details in the text); red line denotes linear fit. **(c–e)** are examples of FFR trials for three levels of grouping: high **(c)**, low **(d)** and intermediate **(e)**. All words consecutively recalled from the presented list are shown as a vertical column with color corresponding to list number and height to the number of words in the sequence. High level of grouping **(c)** is characterized by consecutive recalls of many words from the same list, sometimes interleaved with 1 or 2 words from other lists. Low level of grouping **(d)** is characterized by frequent switches between lists with 1 or 2 words recalled consecutively from the same list. Intermediate level of grouping **(e)** shows a mixture of high and low level grouping.

The protocol of the experimental dataset we analyze, obtained in the lab of Prof. Kahana at the University of Pennsylvania^43^, adheres to the following structure. Each participant performed 16 Immediate Free Recall (IFR) trials a day with randomly assembled non-overlapping lists of 16 words. On selected days they were subsequently asked to recall all the words presented on that day (FFR; Fig. 1a). Averaged over roughly 900 FFR sessions, participants recalled 57 words per session. This level of performance is much higher than typical recall performance of lists of 16×16 = 256 words^2,41^, indicating that participants take advantage of the structural organization of presented words imposed by prior IFR trials. To prove that this is indeed the case, we quantify the level of grouping in FFR over the presented lists with a value *p*_16_ that reflects the tendency to recall subsequent words from the same list before switching to another list (see Materials and Methods)^44^. The distribution of *p*_16_ over the data is very wide (Fig. 1b), covering the range from 0 (random recall) to 0.9 (strong degree of grouping; see Fig. 1c–e for three prototypical examples). Displaying the FFR performance versus the grouping measure *p*_16_ revealed a striking correlation between the two (*r* = 0.62, *p* = 4×10^−97^), with the bulk of data well characterized by linear dependence of performance on *p*_16_. Interestingly, in the limit *p*_16_ → 0, i.e. when no grouping is employed, performance approached a value of 30 words, supporting the theoretical prediction^32^. We also observe that in FFR sessions with highest values of *p*_16_ participants occasionally recalled single words from a list in between longer sequences from other lists (Fig. 1c; see e.g. a single word from the 15^*th*^ list recalled between two groups of words from the 4^*th*^ list). We speculate that these short ‘intrusions’ are analogous to famous ‘slips of the tongue’ in natural speech^45^.

A possible interpretation of the above results is that participants perform FFR by applying a mixture of two recall strategies, one that treats all the words as one long random list, and another one that operates on two levels, namely individually presented lists and words within a list. As the second strategy gains prominence, recall becomes progressively more grouped and the value of *p*_16_ increases, accompanied by the increase in performance. In particular, the participants could develop stable representations of each list as a separate entity and ‘recall’ a list before recalling words from that list.

### Spontaneous grouping within presented lists

**Figure 2:**
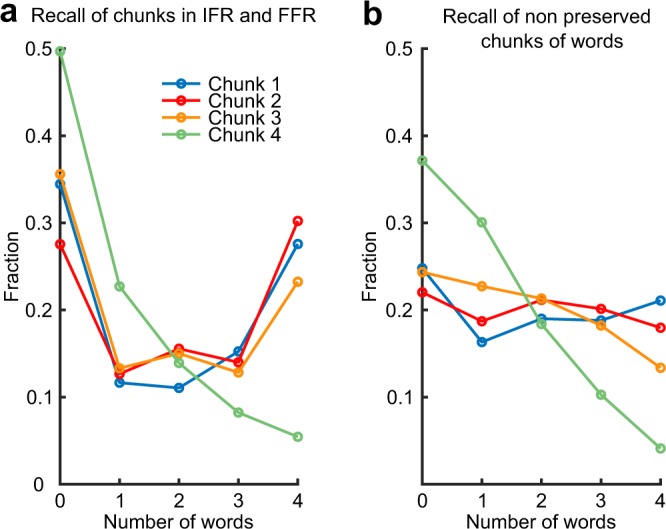
Fraction of chunk pieces of different length (number of words) recalled during final free recall (FFR). **(a)** When chunk is recalled as a whole (all 4 words from the chunk are recalled consecutively) in Immediate Free Recall (IFR). **(b)** When all words from a chunk recalled in IFR, but there are interleaving words from different chunks, i.e. a chunk is not recalled as a whole.

The grouping over lists exhibited in Fig. 1 is induced by the experimental protocol as lists are first presented and recalled individually in the IFR protocol. Another level of grouping, that was not induced by the protocol, was identified in FFR through the analysis of IFR data: a small proportion of participants develops chunking strategies in IFR^46,47^. These participants divide lists of 16 words into chunks of 3 or 4 consecutively presented words (e.g. words 1–4, 5–8, 9–12 and 13–16 in case of chunks of size 4) and recall these chunks as single entities^44^. This kind of chunking is not imposed by the protocol; hence, it must emerge from active manipulation of the presented list, for example representing chunks of words as separate items in memory. Here we wondered whether the chunks observed during IFR remained in memory till FFR trials. It is hard to infer whether chunking occurred in every single trial, hence we assumed that a chunk is recalled as a unit when all words from that chunk are recalled consecutively in IFR (not necessarily in the correct order). We therefore isolated all chunks of size 4 that were recalled during IFR trials (as described above), and considered the recall of the constituting words during FFR. We computed the probability for the different number of words from this chunk to be recalled. The results are shown in Fig. 2a. We found that for the first three chunks in the list, probability has two peaks, at 0 and 4 words, indicating the tendency for all 4 words in these chunks to be recalled or omitted as a single unit. Interestingly, the probability curve for the last chunk in a list decays monotonically, indicating that words from that chunk are recalled independently. A plausible explanation of this effect is that the last several words in a list are typically recalled immediately during IFR since they are maintained in working memory after the list is presented, and hence their recall is effortless and does not lead to the formation of a chunk representation in memory. A similar explanation also accounts for a recently reported ‘anti-recency’ effect in FFR, where the last words in a list have *lower* probability to be recalled, as opposed to the well-documented positive recency effect during IFR^39^. For comparison, if the same analysis is performed for IFR trials where the same four words were recalled but with at least one intervening word, the corresponding probabilities do not exhibit a peak at four words recalled (Fig. 2b).

### Spontaneous grouping of lists

**Figure 3:**
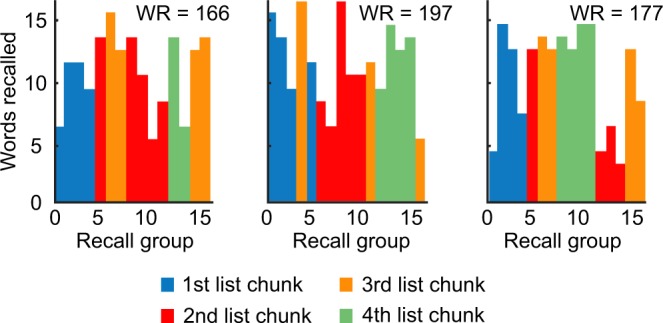
Three examples of FFR sessions with strong list chunking. Each plot displays the structure of a FFR session. Each bar represents a long sequence of words recalled from a single list with the height of the bar indicating the number of words in a sequence and the color corresponding to the list chunk number. On top is reported the number of words recalled (WR) in each of these sessions.

Some of the best participants who employ a strong over the list grouping imposed by the presentation protocol, also exhibit a higher-level grouping of lists. In particular, they tend to recall lists in chunks of four consecutive lists, as illustrated in figure Fig. 3.

Taken together, the results presented above illustrate that our memory is trained to create a structure on different levels of organization, including those that are not directly imposed by the presentation protocol.

### Hierarchical model of memory recall

The model developed for this study generalizes our previous model of single list free recall that is based on two principles^32,40^:

1. The encoding principle states that each memory item is encoded (“represented”) in the brain by a specific group of neurons in a dedicated memory network. When an item is retrieved (“recalled”), either spontaneously or when triggered by an external cue, this specific group of neurons is activated.
2. The associativity principle for which, in the absence of specific retrieval cues, the currently retrieved item plays the role of an internal cue that triggers the retrieval of the next item.

From these two principles we were able to theoretically predict that, out of *L* remembered words, on average, $\sqrt{3\pi L/2}\approx 2.17\sqrt{L}$ words would be recalled^41^. This matches well with the average performance and its distribution in single list recall of approximately 8 words out of 16^32^. However, the average FFR performance of 57 words recalled out of 256 words presented over the entire session (16 lists of 16 words each) is much higher than predicted by this model, which motivated the current extension. To this end, we build an hierarchical model of memory based on these two principles and show that it’s behavior is in agreement with experimental results presented above. Our model could be viewed as a radically simplified version of  ^24^ and^14^.

#### Modeling the encoding

**Figure 4:**
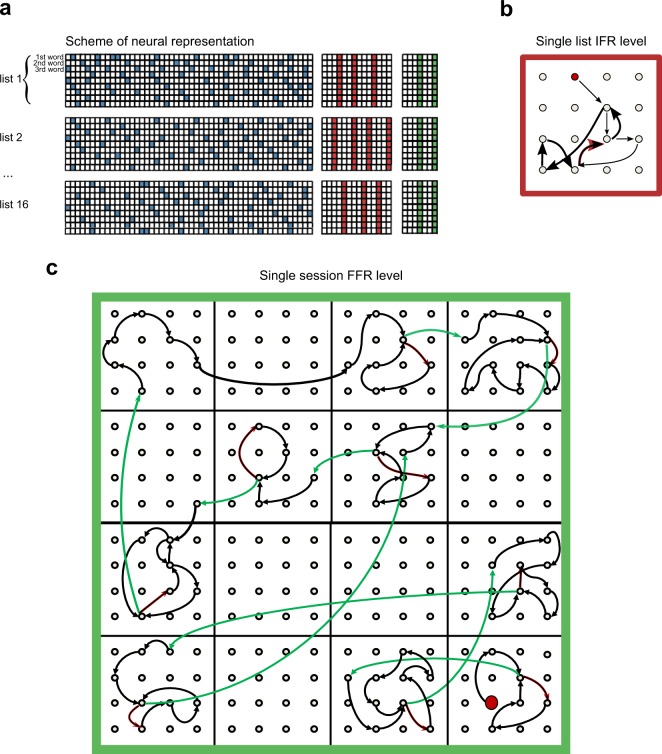
**(a)** Graphical illustration of the encoding of information in the dedicated memory network. 3 subnetworks encoding words, lists and sessions are shown. Each row corresponds to a word and each column to a neuron. Colored neurons encode words (blue color), lists (red color) or sessions (green color). Each word in a word subnetwork is encoded by small fraction of randomly selected neurons, but all words from a given list have the same list and session representations. **(b)** An example trajectory of recall transitions induced by the model during the IFR of a single list of 16 words. Red circle indicates the initial word recalled. The red arrow is the first transition that is repeated, initiating the retrieval cycle that is shown with bold arrows. **(c)** Same for a FFR trial with large binding constant *α*. Structured transitions are shown in black, random transitions are shown in green. Red arrows show repeated within-list transitions that, if taken, would result in a cycle, as in (**b**). In the FFR model they are not taken, rather they trigger the suppression of list representations and initiation of random transitions shown in green (see details in text). Red circle is an initial item recalled.

We extend the encoding principle formulated above for the recall of single lists in the following way. Following^14,24^ we postulate that different distinct levels of information (words, chunks, lists, context…) are encoded in the form of sparse random neuronal populations in the corresponding distinct subnetworks (see Fig. 4a). In the experimental paradigm words are presented in lists of 16 items and each session consists of 16 lists. Accordingly, each word *W* is labeled by the triple of indexes: *W* = (*w*, *l*, *s*), corresponding to the presentation position of the word in the session (from 1 to 256), the presentation position of the list (from 1 to 16), and the session number, respectively. Similar to CMR^14^, we represent each word *W* by the concatenation of three binary patterns, each representing a session, list with a session an a word within a list, respectively:

1 $${{\xi }}^{W}=\mathop{\underbrace{010010001\ldots 10}}\limits_{{\rm{w}}{\rm{o}}{\rm{r}}{\rm{d}}\,{\rm{e}}{\rm{n}}{\rm{c}}{\rm{o}}{\rm{d}}{\rm{i}}{\rm{n}}{\rm{g}}\,{{\xi }}^{{\rm{w}}}}\mathop{\underbrace{010010100\ldots 10}}\limits_{{\rm{l}}{\rm{i}}{\rm{s}}{\rm{t}}\,{\rm{e}}{\rm{n}}{\rm{c}}{\rm{o}}{\rm{d}}{\rm{i}}{\rm{n}}{\rm{g}}\,{{\xi }}^{{\rm{l}}}}\mathop{\underbrace{101011000\ldots 1}}\limits_{{\rm{s}}{\rm{e}}{\rm{s}}{\rm{s}}{\rm{i}}{\rm{o}}{\rm{n}}\,{\rm{e}}{\rm{n}}{\rm{c}}{\rm{o}}{\rm{d}}{\rm{i}}{\rm{n}}{\rm{g}}\,{{\xi }}^{{\rm{s}}}}$$

The length of the three vectors equals the number of neurons in each subnetwork *N*_*w*_, *N*_*l*_, *N*_*s*_. Each neuron contributes to the encoding of a word *W* with probability *f* so that the total number of neurons which encode a word *W* is on average *f*⋅(*N*_*w*_ + *N*_*l*_ + *N*_*s*_) = *f*⋅*N*.

In our previous studies^32,48^, transitions between words were driven by similarity defined as a dot product between the corresponding representations (see Methods). Due to the decomposition of representations into three parts (see Eq. (1)), the similarity between any two words *W*_1_ and *W*_2_ can be presented as a sum of three corresponding terms:

2 $${S}_{tot}^{{W}_{1},{W}_{2}}={S}_{word}^{{w}_{1},{w}_{2}}+\alpha {S}_{list}^{{l}_{1},{l}_{2}}+\beta {S}_{session}^{{s}_{1},{s}_{2}},$$

where ${S}_{word}^{{w}_{1},{w}_{2}}$ is the similarity matrix between words *w*_1_ and *w*_2_ in words subnetwork; ${S}_{list}^{{l}_{1},{l}_{2}}$ is the similarity between lists *l*_1_ and *l*_2_, to which the words *W*_1_ and *W*_2_ belong; ${S}_{session}^{{s}_{1},{s}_{2}}$ is the similarity of sessions *s*_1_ and *s*_2_ in the session subnetwork; parameters *α* and *β* weight the relative strength of the list and session context populations respectively in driving the retrieval process.

The data shows that IFR had a strong effect on FFR, since, while average IFR performance was 50%, 87% of the words recalled in FFR are the words that were previously recalled in IFR. We therefore assumed that only the words that are recalled during IFR are bound to list and session representations, i.e. the last two terms in the total similarity matrix of Eq. (2) are only added for pairs of words that were both recalled during IFR (see Methods).

#### Associative transitions

The model of the encoding principle provides a simple mathematical characterization of words representation, but it does not describe how these representations are exploited in the retrieval dynamics. This is described within the scope of the associative principle which determines transitions between words.

According to the associativity principle the currently retrieved item functions as an internal cue that triggers the retrieval of the next one. Transitions between words are brought about by similarities between the active word – the last retrieved one – and other encoded words. Simplifying the previous models^14,24^, we use the deterministic transition rule, namely, the word which is most similar to the currently retrieved one is then activated and the process continues leading to the retrieval of more and more words. Importantly, the last retrieved word cannot be activated so that a transition which just occured cannot immediately happen in the reverse direction. The IFR of a single list was obtained by the non-hierarchical recall model of^40^ with word-to-word contribution *S*_*word*_ to the similarity matrix of Eq. (2) (see Fig. 4b). The recalled words in IFR were then used to build the total similarity matrix *S*_*tot*_ of Eq. (2) for different strengths of binding between words and lists, *α,* and FFR was modeled as follows. The dynamical recall process is driven by *S*_*tot*_ (see Fig. 4c, black arrows), unless the same within-list transition is attempted for the second time (Fig. 4b,c, red arrows). At this point, the process, if continued, would enter a loop by recapitulating the same words of a given list that were already retrieved and hence no new words would be recalled (Fig. 4b, bold arrows). Note that the repeated retrieval of the same word not always initiates the loop because sometimes the retrieval could then proceed in the opposite direction (see^40^ and Fig. 4b). Similar to^42^, we assume than when the process approaches a loop, i.e. the same within-list transition is attempted for the second time, the list representation is suppressed and the next transition is determined by the other two contributions in the similarity matrix corresponding to session context and word-to-word similarity:

3 $${S}_{tot-l}^{{W}_{1},{W}_{2}}={S}_{word}^{{w}_{1},{w}_{2}}+\beta {S}_{session}^{{s}_{1},{s}_{2}}.$$

We call these transitions ‘random’ in contrast to the ‘structured’ ones induced by *S*_*tot*_, and show them with green arrows in Fig. 4c. Upon triggering the retrieval of a new word through random transition the process reverts to using the full similarity matrix *S*_*tot*_ with the list representation corresponding to the retrieved word activated, until it eventually enters a big loop that includes several lists.

#### Comparison between data and model simulation

**Figure 5:**
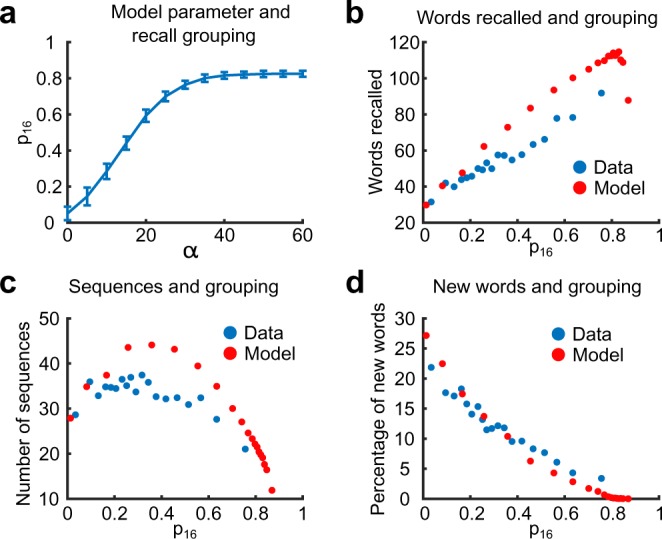
Hierarchical model results. **(a)** Value of the induced *p*_16_ in the model simulations as a function of *α*. Blue line is the average of the value of *p*_16_ with a confidence interval of 95%. **(b)** Scatter plot of the performance as a function of *p*_16_ both data and model. For each session the number of words retrieved in FFR is shown against the *p*_16_ score. **(c)** Scatter plot for the number of sequences retrieved as a function of *p*_16_ for each session both data and model. **(d)** Scatter plot for the percentage of words not retrieved in the IFR but recalled in the FFR as a function of *p*_16_ for each session both data and model.

We now turn to deploying this model in simulating the experimental paradigm analyzed previously. To qualitatively compare the model to experimental findings, we examine how the sequences generated by our model present grouping of items as measured by *p*_16_. In the model, the parameter *α* controls the strength of binding between the words recalled during IFR and the list representation. When *α* is high, the similarity between the words from the same list is high and hence most of the transitions happen between such words. We let *α* vary across sessions, and set the binding between words and sessions according to $\beta =\frac{\alpha }{2}+\gamma$. Here *γ* is a constant that controls the binding of words recalled during IFR to a session, irrespective of how strong the list binding is. The reason for this contribution is that the words recalled in IFR have a higher chance to be recalled in FFR even for sessions with no list grouping (i.e. sessions with *p*_16_ = 0), see Fig. 5d. The exact relation between *β* and *α* is not important, besides setting the value of *α* for which grouping saturates (see Fig. 5a).

Using the described model, 6500 sessions of FFR were simulated. We compute *p*_16_ for all sessions of FFR so generated and find that *p*_16_ on average monotonically increases with the value of *α*, Fig. 5a. This is an expected behavior since large values of *α* force structured recall. Similarly to experimental data the model shows a linear dependence of the number of recalled words as a function of *p*_16_, Fig. 5b (cfr. Fig. 1b). Intriguingly, the number of sequences of words recalled from the same list as a function of *p*_16_ shows a non-monotonic dependence, Fig. 5c (red dots), which we also observed in the experimental data (blue dots). For small values of *α*, and thus *p*_16_, the recall is unstructured and the number of sequences is roughly equal to the number of recalled words (see Fig. 1d). When *α* and, therefore, *p*_16_ increases the number of sequences increases since there is a mixture of two recall processes - random and structured (see Fig. 1e). For intermediate values of *p*_16_ the contribution of *S*_*word*_ and *S*_*list*_ to driving structured transitions are comparable and across lists transitions may still be triggered by structured transitions. As we further increase *α* the recall becomes very structured and the words from a single list are predominantly recalled before words from other lists are recalled (see Fig. 1c). Consequently the number of sequences becomes comparable, or even smaller than the number of presented lists. To further assess the validity of our model we compute the percentage of newly recalled words in FFR (the words that were not recalled in IFR). Figure 5d shows that this steadily decreases with *p*_16_ for both the model (red dots) and the experimental data (blue dots).

## Discussion

We studied the final free recall of sets of 256 unrelated words that were previously presented and recalled on the same day as 16 lists of 16 words each. We found that FFR trials exhibit various degrees of hierarchical organization: within-list chunking that spontaneously emerged in IFR, over-the-list organization induced by the presentation protocol, and finally list chunking for the very best participants (see Figs 1–3 above). The dominant recall organization, exhibited in the bulk of the data, was the tendency to recall subsequent words from the same list. This type of grouping strongly correlated with performance, Fig. 1b. When extrapolated to the limit of random recall, the performance dipped below the level of 30 words that closely matched our theoretical prediction for structure-less recall. The average performance was almost twice higher than this level, indicating a strong effect of information structure on memory retrieval. We also found that within-list chunks that emerged spontaneously in a limited number of trials in IFR^44^ have a high probability to be recalled or omitted as single units during FFR trials as well. Taken together, our results strongly indicate that people tend to organize information to be remembered in a way that facilitate subsequent recall, even when information itself lacks any meaning, as in the case of free recall of random words.

From a theoretical point of view, we extended the model of associative memory recall^40^ to take into account the hierarchical representation of information in FFR that we found in experimental data. More specifically, we added list and session context subnetworks. The resulting model is compatible with proposed principles of sparse encoding and associative transitions. Our model is much simpler than the previous models of hierarchical contextual recall^14,24^, which helps to better understand the relation between the binding of memorized words to context and FFR properties. It should be noted however that the simplicity of the model comes with the price, since it does not account for temporal organization of single list recall, such as primacy, recency and contiguity. Our experimental and theoretical results indicate that the recall of the words in IFR, rather than passive acquisition, is a dominating factor in the emergence of the grouping. The model can be easily generalized to any number of hierarchical levels by adding additional layers of representations, similar to list and session representations.

## Methods

### Experimental methods

Similarly to^49^, the data reported in this manuscript were collected in the lab of M. Kahana as part of the Penn Electrophysiology of Encoding and Retrieval Study (see^43^ for details of the experiments). Here we analyzed the results from the 217 participants (age 17–30) who completed the first phase of the experiment, consisting of 7 experimental sessions. All experiments were performed in accordance with relevant guidelines and regulations. Participants were consented according the University of Pennsylvania’s IRB protocol and were compensated for their participation. Informed consent was obtained from all participants or, if participants are under 18, from a parent or legal guardian. Each session consisted of 16 lists of 16 words presented one at a time on a computer screen and lasted approximately 1.5 hours. Each study list was followed by an immediate free recall test. Words were drawn from a pool of 1638 words. For each list, there was a 1500 ms delay before the first word appeared on the screen. Each item was on the screen for 3000 ms, followed by jittered 800–1200 ms inter-stimulus interval (uniform distribution). After the last item in the list, there was a 1200–1400 ms jittered delay, after which participants were given 75 seconds to attempt to recall any of the just-presented items. In 4 out of 7 experimental sessions, following the immediate free recall test from the last list, participants were shown an instruction screen for final-free recall, informing them to recall all the items from the preceding lists in any order. After a 5 s delay, a tone sounded and a row of asterisks appeared. Participants had 5 minutes to orally recall any item from the preceding lists.

### Grouping measures

For each final-free recall trial we consider the ordered set of recalled words (*W*) defined as *w*_1_ → *w*_2_ → … → *w*_*n*_ where *n* is the number of words recalled in a given trial and *w*_1_ (*w*_2_, …, *w*_*n*_) denotes the input serial position during the day of the first (second, …, last) word recalled, which is the number between 1 and 256 (see Fig. 1a). We introduce the grouping measure (*p*), and assign the probability to each transition by assuming that the next word recalled is chosen from the same list as the currently recalled word with probability p and a random word is chosen with probability 1 − *p*. The probability for the whole sequence is computed as a product of individual transition probabilities. Formally, if *l*_*i*_ is the number of the list (from 1 to 16) from which word *w*_*i*_ was presented, the probability *P*_*i*_ of transition (*w*_*i*_ → *w*_*i*+1_) and the total logarithm probability of the whole sequence (log-likelihood) are

4 $$\begin{array}{rcl}{P}_{i} & = & \{\begin{array}{ll}\frac{p\delta ({l}_{i+1},{l}_{i})}{{m}_{i}}+\frac{1-p}{L-i} & {m}_{i} > 0\\ \frac{1}{L-i} & {m}_{i}=0\end{array}\\ l(W|p) & = & \sum _{i=1}^{n-1}\,\mathrm{log}\,({P}_{i})\end{array}$$

where *m*_*i*_ ∈ [0, …, 15] is the number of not yet recalled words from the list *l*_*i*_ ∈ [1, …, 16] and *L* = 256 is the total number of words presented during the day. The grouping measure *p*_16_ for the FFR trial is then obtained as the value of *p* that maximizes the likelihood of the sequence *l*(*W*|*p*).

### Theoretical model details

The model builds on the idea that words are represented as binary population vectors, Eq. (1). The full similarity matrix between two words is then given by Eq. (2). Given two words *W*_1_ and *W*_2_ the contributions of the different terms is given by

5 $$\begin{array}{rcl}{S}_{word}^{{w}_{1},{w}_{2}} & = & \sum _{i=1}^{{N}_{w}}\,{\xi }_{i}^{{w}_{1}}{\xi }_{i}^{{w}_{2}}\simeq (\begin{array}{cc} {\mathcal B} ({N}_{w},f), & {w}_{1}={w}_{2}\\  {\mathcal B} ({N}_{w},{f}^{2}), & {w}_{1}\ne {w}_{2}\end{array}\\ {S}_{list}^{{l}_{1},{l}_{2}} & = & \frac{1}{{N}_{l}\,f}{\rm{IFR}}({W}_{1}){\rm{IFR}}({W}_{2})\cdot \sum _{i\mathrm{=1}}^{{N}_{l}}\,{\xi }_{i}^{{l}_{1}}{\xi }_{i}^{{l}_{2}}\\  & \simeq  & \frac{1}{{N}_{l}\,f} {\mathcal B} ({N}_{l},f)\cdot {\rm{IFR}}({W}_{1}){\rm{IFR}}({W}_{2})\cdot {\delta }_{{l}_{1},{l}_{2}}\\ {S}_{session}^{{s}_{1},{s}_{2}} & = & \frac{1}{{N}_{s}\,f}{\rm{IFR}}({W}_{1}){\rm{IFR}}({W}_{2})\cdot \sum _{i=1}^{{N}_{s}}\,{\xi }_{i}^{{s}_{1}}{\xi }_{i}^{{s}_{2}}\\  & \simeq  & {\rm{IFR}}({W}_{1}){\rm{IFR}}({W}_{2})\cdot {\delta }_{{s}_{1},{s}_{2}}\end{array}$$

where *w*_1_, *w*_2_ index the word coding part of the population vector *ξ*, *l*_1_, *l*_2_ the list coding part, *s*_1_, *s*_2_ the session context coding part, while *IFR*(*W*) is an indicator function that word *W* was recalled in the IFR trial following the list presentation, i.e. *IFR*(*W*) = 1 was retrieved and 0 otherwise. To speedup simulations we neglected the correlations between elements in similarity matrices and approximate them by independent binomial random variables ${\mathcal B} ({N}_{w},{f}^{2})$ for word similarities and ${\mathcal B} ({N}_{l},f)$ for list similarities. We neglected similarities between different lists and different sessions and also assumed session similarities to be equal to each other. The list and session similarity matrices were normalized to have entries of the order of 1.

According to the associative principle, given an active word *W*_*k*_ the formal equation that defines the next word retrieved during structured recall is

6 $${W}_{k+1}=\mathop{{\rm{argmax}}}\limits_{W\notin {M}_{k}}\,{S}_{tot}^{{W}_{k},W},$$

where *M*_1_ = {*W*_1_}, and *M*_*k*_ = {*W*_*k*−1_, *W*_*k*_}. Similarly for random transitions

7 $${W}_{k+1}=\mathop{{\rm{a}}{\rm{r}}{\rm{g}}{\rm{m}}{\rm{a}}{\rm{x}}}\limits_{W\notin {M}_{k}}\,{S}_{tot-l}^{{W}_{k},W}$$

where *S*_*tot*−*l*_ is given by Eq. (3). In the simulations we consider *N* = 300000 and *f* = 0.1, ${N}_{w}=\frac{N}{3}$, ${N}_{l}=\frac{N}{3}$, ${N}_{s}=\frac{N}{3}$, *γ* = 15. This value for *γ* was chosen to match the proportion of new words recalled during FFR on sessions with little over the list grouping (see Fig. 5d).
